# Treatment of MAS and HLH

**DOI:** 10.1186/1546-0096-12-S1-I8

**Published:** 2014-09-17

**Authors:** Alexandre Belot

**Affiliations:** 1 Pediatric Nephrology, Rheumatology and Dermatology, Hopital Femme Mère Enfant, Hospices Civils de Lyon, France; 2 U1111, INSERM, Lyon, France

## 

Macrophage activation syndrome (MAS) is a life-threatening complication of inflammatory disease, occurring secondary to a complex interplay of genetic factors, drugs, infectious agents and immunological anomalies. Early identification and aggressive treatment are mandatory to prevent fatal evolution.

Precipitating factors should be looked for and eventually removed such as infections (leishmania, EBV, Parvo B19…) or drug exposure. Epstein Barr Virus (EBV) is a major cause of MAS and anti-EBV therapies can be helpful to control MAS.

First line therapies usually include high-dose steroids associated to cyclosporine. In the context of primary hemophagocytic lymphohistiocytosis (HLH), bone marrow transplantation is the only treatment able to cure the disease. In inflammatory disease with secondary HLH, a few case reports indicate an efficacy of anti-cytokine treatment (anti-IL1, anti-IL6, anti-TNFα). However, a role of these cytokines in MAS development remains unproven. To investigate whether the IL-1 pathway might contribute to MAS, we compared IL-1RA-/- to wild type mice after stimulation with CpG, a TLR9 activator. TLR9-induced MAS was similar in the two groups, suggesting that IL-1 excess is not a major inducer of MAS. More interestingly, recent data implicate IFNγ as a crucial factor in MAS onset. Thus, the inhibition of secreted IFNγ might represent an interesting therapeutic avenue worthy of further investigation.

## Disclosure of interest

None declared.

